# Unexpectedly Higher Morbidity and Mortality of Hospitalized Elderly Patients Associated with Rhinovirus Compared with Influenza Virus Respiratory Tract Infection

**DOI:** 10.3390/ijms18020259

**Published:** 2017-01-26

**Authors:** Ivan F. N. Hung, Anna Jinxia Zhang, Kelvin K. W. To, Jasper F. W. Chan, Shawn H. S. Zhu, Ricky Zhang, Tuen-Ching Chan, Kwok-Hung Chan, Kwok-Yung Yuen

**Affiliations:** 1 State Key Laboratory for Emerging Infectious Diseases, Carol Yu’s Centre for Infection and Division of Infectious Diseases, The University of Hong Kong, Queen Mary Hospital, Hong Kong SAR, China; zhangajx@hku.hk (A.J.Z.); kelvinto@hku.hk (K.K.W.T.); jaspchan@gmail.com (J.F.W.C.); houshunzhu@gmail.com (S.H.S.Z.); rickychang87@gmail.com (R.Z.); chankh2@hku.hk (K.-H.C.); kyyuen@hku.hk (K.-Y.Y.); 2 Department of Medicine, The University of Hong Kong, Queen Mary Hospital, 102 Pokfulam Road, Pokfulam, Hong Kong SAR, China; tuenching@yahoo.com.hk

**Keywords:** rhinovirus, influenza, hospitalized, elderly, mortality, respiratory, tract

## Abstract

Rhinovirus is a common cause of upper and lower respiratory tract infections in adults, especially among the elderly and immunocompromised. Nevertheless, its clinical characteristics and mortality risks have not been well described. A retrospective analysis on a prospective cohort was conducted in a single teaching hospital center over a one-year period. We compared adult patients hospitalized for pneumonia caused by rhinovirus infection with those hospitalized for influenza infection during the same period. All recruited patients were followed up for at least 3 months up to 15 months. Independent risk factors associated with mortality for rhinovirus infection were identified. Between 1 March 2014 and 28 February 2015, a total of 1946 patients were consecutively included for analysis. Of these, 728 patients were hospitalized for rhinovirus infection and 1218 patients were hospitalized for influenza infection. Significantly more rhinovirus patients were elderly home residents and had chronic lung diseases (*p* < 0.001), whereas more influenza patients had previous stroke (*p* = 0.02); otherwise, there were no differences in the Charlson comorbidity indexes between the two groups. More patients in the rhinovirus group developed pneumonia complications (*p* = 0.03), required oxygen therapy, and had a longer hospitalization period (*p* < 0.001), whereas more patients in the influenza virus group presented with fever (*p* < 0.001) and upper respiratory tract symptoms of cough and sore throat (*p* < 0.001), and developed cardiovascular complications (*p* < 0.001). The 30-day (*p* < 0.05), 90-day (*p* < 0.01), and 1-year (*p* < 0.01) mortality rate was significantly higher in the rhinovirus group than the influenza virus group. Intensive care unit admission (odds ratio (OR): 9.56; 95% confidence interval (C.I.) 2.17–42.18), elderly home residents (OR: 2.60; 95% C.I. 1.56–4.33), requirement of oxygen therapy during hospitalization (OR: 2.62; 95% C.I. 1.62–4.24), and hemoglobin level <13.3 g/dL upon admission (OR: 2.43; 95% C.I. 1.16–5.12) were independent risk factors associated with 1-year mortality in patients hospitalized for rhinovirus infection. Rhinovirus infection in the adults was associated with significantly higher mortality and longer hospitalization when compared with influenza virus infection. Institutionalized older adults were particularly at risk. More stringent infection control among health care workers in elderly homes could lower the infection rate before an effective vaccine and antiviral become available.

## 1. Introduction

Human rhinoviruses (HRV) are RNA virus from the Picornaviridae family and are divided into three species A, B, and C [1,2,3,4]. Currently, more than 100 distinct serotypes have been identified. Every year, these viruses cause both upper and lower respiratory tract infections in young children and adults during the spring and fall period in Hong Kong [5,6]. Clinical presentations varied from asymptomatic infection, to upper respiratory symptoms including rhinorrhea, postnasal drip, and cough, and to the more severe trachea-bronchitis and pneumonia, requiring hospitalization [1,7,8]. Older adults and immunocompromised patients were particularly at risk of developing severe infection and bacterial co-infection, requiring prolonged hospitalization and treatment [8,9]. Patients with underlying chronic obstructive pulmonary disease (COPD) and asthma are also at risk of developing acute exacerbations [10,11]. HRV is predominantly transmitted via droplets or by indirect contact with contaminated fomites. Major rhinovirus outbreaks in health care facilities have been reported [12,13,14,15,16].

Despite the clinical importance of HRV infection, the clinical characteristics and mortality risk factors have not been well described. The objective of the current retrospective analysis on a prospective cohort was to compare adult patients hospitalized for HRV infection with those hospitalized for influenza infection during the same period and to identify independent risk factors associated with mortality for HRV infection.

## 2. Results

**Figure 1:**
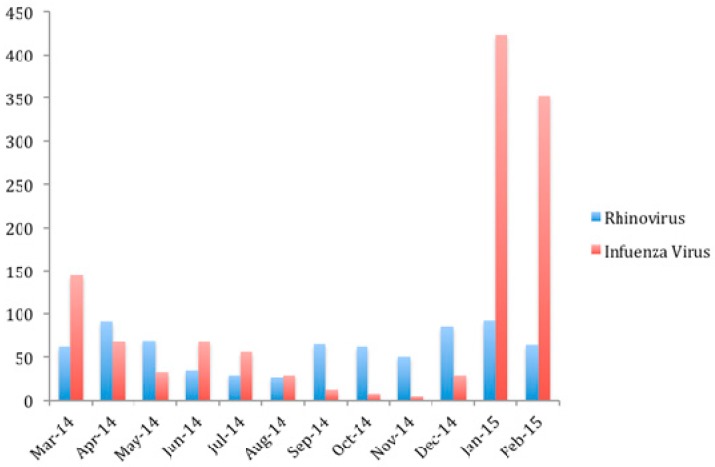
Cases of rhinovirus and influenza hospitalized in Queen Mary Hospital between 1 March 2014 and 28 February 2015.

Between 1 March 2014 and 28 February 2015, a total of 1946 patients were recruited consecutively (Table 1). Of these, 728 patients were hospitalized for HRV infection, and 1218 patients were hospitalized for influenza infection. Among those patients admitted for influenza infection, 76.5% was H3N2, 3.8% was H1N1, 19.3% was B, and 0.4% was C. The overall mean age was 72.9 years (Standard deviations (S.D.): 19.2 years) and 44.7% were male. Majority of the influenza cases (63.6%) was admitted during the winter peak in January and February 2015 (Figure 1), with a smaller summer peak (10.1%) in June and July 2014. Cases of HRV were admitted evenly throughout the year, with fewer cases between June and August 2014.

Comparing the HRV group with the influenza group (Table 1), significantly more HRV patients were elderly home residents and had chronic pulmonary diseases (*p* < 0.001), whereas more influenza patients had previous stroke (*p* = 0.02); otherwise, there were no differences in the Charlson comorbidity indexes between the two groups. More patients in the rhinovirus group presented with chest wheezes (*p* = 0.007), developed pneumonia complication (*p* = 0.03), required oxygen therapy, and had a longer hospitalization period (*p* < 0.001), whereas more patients in the influenza virus group presented with fever (*p* < 0.001) and upper respiratory tract symptoms of cough (*p* < 0.001), sore throat (*p* < 0.001) and rhinorrhea (*p* = 0.001), developed cardiovascular (*p* < 0.001) and sepsis complications (*p* = 0.03).

Comparing the laboratory findings upon admission between the HRV and influenza groups (Table 1), the HRV group has a significantly higher proportion of patients with hemoglobin <13.3 g/dL (*p* = 0.01), neutrophil count >7.42 × 10^9^ /L (*p* < 0.001) and creatinine kinase >174 IU/L (*p* = 0.01) compared with the influenza group, which has a significantly higher proportion of patients with AST >38 IU/L (*p* < 0.001). Concerning the bacteriological findings, there were no differences in the culture positive specimens between the two groups. Sputum culture was positive for *Streptococcus pneumoniae* (1% vs. 0.5%; *p* = 0.22), *Haemophilus influenzae* (HRV vs. influenza) (2.1% vs. 1.9%; *p* = 0.79), *Staphylococcus aureus* (1.5% vs. 1.1%; *p* = 0.49), *Pseudomonas aeruginosa* (2.2% vs. 2.1%; *p* = 0.93), *Klebsiella species* (1% vs. 0.8%; *p* = 0.74), *Acinetobacter baumannii* (0.1% vs. 0.3%; *p* = 0.42), and *Moraxella catarrhalis* (0.3% vs. 0.2%; *p* = 0.91). Blood culture was positive for *Streptococcus pneumoniae* (0.3% vs. 0.2%; *p* = 0.6), *Staphylococcus aureus* (0.1% vs. 0.08%; *p* = 0.71), *Proprionibacterium acnes* (0% vs. 0.2%; *p* = 0.27), *Escherichia coli* (0.2% vs. 0.1%; *p* = 0.61), *Acinetobacter baumannii* (0.1% vs. 0.2%; *p* = 0.88), *Moraxella catarrhalis* (0.1% vs. 0%; *p* = 0.2), *Morganella morganii* (0% vs. 0.1%; *p* = 0.44), *Proteus species* (0.1% vs. 0%; *p* = 0.2), *Enterococcus faecalis* (0.1% vs. 0%; *p* = 0.2), *Bacteriodes species* (0% vs. 0.1%; *p* = 0.44), and *Citrobacter freundii* (0% vs. 0.1%; *p* = 0.44).

Comparing the radiological findings upon admission between the HRV and influenza groups, majority of the patients (21.8%) in both groups presented with right lower zone opacity or consolidation on chest radiograph taken upon admission. Significantly more patients in the HRV group presented with right upper zone (5.9% vs. 2.4%), right middle zone (3.2% vs. 0.7%), left upper zone (3.3% vs. 0.2%), and bilateral upper zone (2.7% vs. 0.2%) opacity or consolidation (*p* < 0.001), whereas more influenza patients presented with left lower zone (3.5% vs. 1.5%) or bilateral lower zone (3.4% vs. 0.7%) lesions (*p* < 0.001).

**Figure 2:**
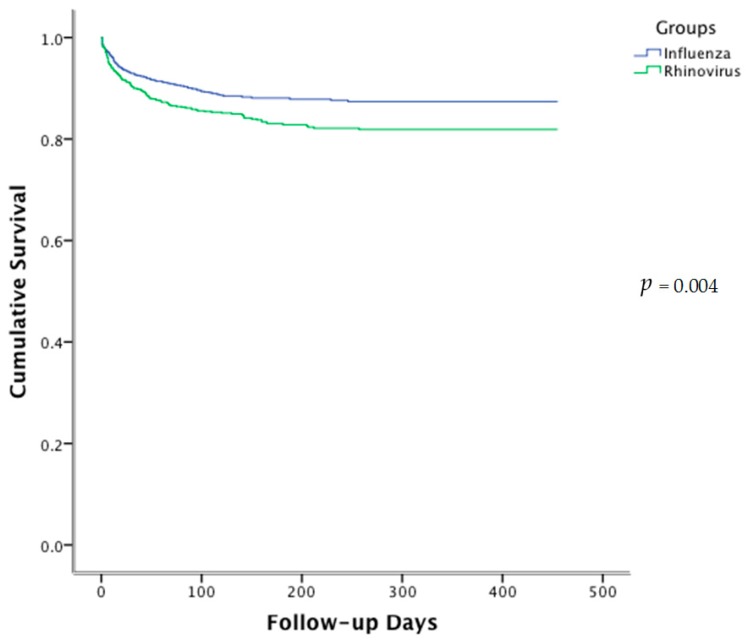
Kaplan–Meier survival curve of patients hospitalized for rhinovirus and influenza infection.

The 30-day (*p* = 0.04), the 90-day (*p* = 0.006) and 1-year (*p* = 0.004) mortality rate was significantly higher in the HRV group than the influenza virus group (Table 1 and Figure 2). One hundred and twenty-five patients (17.2%) in the HRV group succumbed by the end of the 1-year follow-up, comparing to 143 patients (11.7%) in the influenza group. Majority of the HRV patients died of pneumonia (81.6%), followed by COPD (20.8%), and malignancy (10.4%). A significant proportion of patients had more than one causes of death. There was no significant difference in the cause of death between the two groups.

Comparing patients who succumbed with those who survived in the HRV group by univariate analysis (Table 2), patients in the succumbed group were significantly older (*p* < 0.001) and more patients were elderly home residents (*p* < 0.001). Succumbed patients had a higher Charlson comorbidity index (*p* = 0.04) with more chronic pulmonary diseases (*p* = 0.008). Patients who survived have significantly more upper respiratory tract symptoms including sore throat (*p* = 0.004) and rhinorrhea (*p* = 0.001). Significantly more patients in the succumbed group developed pneumonia (*p* < 0.001), cardiovascular (*p* = 0.008) and sepsis (*p* = 0.03) complications, required oxygen therapy (*p* < 0.001), required non-invasive ventilation (*p* = 0.003) and had longer (*p* < 0.001) and more frequent hospitalization (*p* = 0.02). Patients who succumbed also had, in a significantly higher proportion, hemoglobin <13.3 g/dL (*p* = 0.001), neutrophil count >7.42 × 10^9^ /L (*p* = 0.03), creatinine >109 μmol/L (*p* = 0.03), and AST >38 IU/L (*p* = 0.003) compared with patients who survived.

Multivariate analysis (Table 3) demonstrated that intensive care unit admission (odds ratio (OR): 9.56; 95% confidence interval (C.I.) 2.17–42.18), elderly home residents (OR: 2.60; 95% C.I. 1.56–4.33), the requirement of oxygen therapy during hospitalization (OR: 2.62; 95% C.I. 1.62–4.24), and hemoglobin level <13.3 g/dL upon admission (OR: 2.43; 95% C.I. 1.16–5.12) were independent risk factors associated with 1-year mortality in patients hospitalized for HRV infection. Rhinorrhea was independently associated with lower 1-year mortality (OR: 0.31; 95% C.I. 0.11–0.86).

## 3. Discussion

We reported findings of the first retrospective cohort study comparing hospitalized patients with HRV infection against those with influenza virus infection during the same period. Despite similar baseline demographics and the Charlson comorbidity score, significantly more patients hospitalized for HRV infection were elderly home residents, which was also one of the independent risk factors associated with 1-year mortality. Major HRV outbreaks in health care facilities resulted in severe infections, and death has been reported [12,13,14,15,16]. Household transmission of HRV infection was 1 case per person among 17 siblings [17]. Droplet transmissions in a confined area such as elderly homes and indirect contact with contaminated fomites by health care workers were the most significant culprit [18]. HRV can survive on environmental surfaces for several hours thereby facilitating transmission. Compared to the influenza virus, HRV is a non-enveloped virus, which is difficult to disinfect and with no vaccine and antiviral treatment. Studies have demonstrated that ethanol-containing disinfectant were unable to remove HRV [19,20] and did not reduce HRV infection or related common cold illnesses. Washing with soap and water was more efficient. Therefore, stringent infection control, such as regular hand washing with soap, wearing of surgical mask, and regular cleansing of the air filters, should reduce the risk of major outbreak among elderly home residents.

HRV infections are well known to be associated with acute exacerbation of chronic respiratory disease including asthma, COPD, sinusitis, and otitis media [10,11,21]. It is the most common isolated virus in acute exacerbation of COPD. Therefore, it was not surprising to find that patients hospitalized with HRV infection had significantly more chronic pulmonary diseases when compared to patients hospitalized for influenza infection. HRV patients are at a higher risk of developing pneumonia, and upper respiratory symptoms were not as common as influenza infection. An in vivo study demonstrated that HRV infection increased cytokine and chemokine levels, especially IL-6, which may mediate lower airway symptoms during acute exacerbation of COPD [22]. HRV replication also induces the production of interferon-γ inducible protein 10, which plays an important role in the pathogenesis in COPD exacerbation. Apart from chronic lung diseases, patients with underlying malignancy are also at risk of acquiring HRV infection [23]. Both HRV and influenza patients are at risk of developing acute myocardial infarction and stroke complications [24].

It was interesting to demonstrate differences in radiological findings between patients infected with the two viruses. Significantly more HRV patients presented with upper and middle zone lesions, whereas influenza patients presented with more lower zone lesions. Such differences in distribution could be related to the smaller size (50 nm) and lighter weight of the HRV when compared to the influenza virus (80–120 nm). Besides, significantly more HRV patients had underlying pulmonary diseases, which could affect the initial radiological presentation. The exact reasons required further investigation. Similarly, there were significant differences in the various biochemical markers, including lymphocyte count, haemoglobin, ALT, AST, and creatinine level, between the HRV and influenza groups. Nevertheless, such differences were very small and the data has to be interpreted with caution.

Chronic pulmonary disease and bacterial co-infection in patients with HRV infection could explain the lower hemoglobin level and higher neutrophil count upon admission. Bacterial co-infection also worsened the FEV-1 in COPD patients [25]. The poor lung function coupled with the pneumonia complications led to longer hospital stay and more frequent hospitalization. As a result, HRV infection was associated with significantly higher 30-day, 90-day, and 1-year mortality rates than influenza infection. It was also interesting to find that patients who survived after HRV infection had significantly more common upper respiratory symptoms including rhinorrhea and sore throat upon presentation. In contrast, succumbed patients presented with more prominent lower respiratory symptoms including sputum production and subsequent pneumonia complication. This could be explained by the upregulation of cell surface markers and activation of cytokine and chemokine in the tracheobronchial tree, resulting in early activation of Toll-like receptor 3 (TLR3), thus establishing an effective antiviral response in the upper airways, thereby preventing pneumonia development [26]. Failure to eliminate the rhinovirus in the upper airway resulted in IL-6, IL-4, and IFN-γ production by CD4 cells in the lower airway, causing airway hyperreactivity and lung inflammation [22].

Various anti-HRV agents have undergone clinical trials. Capsid-binding anti-HRV agent pleconaril showed that early treatment exerted antiviral effects and reduced the duration of HRV cold [27]. Nevertheless, pleconaril has not been approved for clinical use due to potential drug interaction. Another oral capsid-binding anti-HRV agent vapendavir showed dose-related antiviral effects with significantly lower upper respiratory symptom scores early in the illness and less frequent HRV RNA detection rate on Day 3 after treatment [28]. Intranasal recombinant interferon-α 2b was shown to be effective in preventing HRV colds as post-exposure prophylaxis but lack treatment effect, whereas inhaled interferon-β has demonstrated therapeutic efficacy in asthmatic patients [29]. Limited antigenic cross-reactivity poses challenges in developing an effective HRV vaccine. Peptide immunogens and T cell inducing vaccine strategies have the greatest potential in providing cross-reactivity against HRV infection [30].

## 4. Materials and Methods

Consecutive patients aged ≥18 years with HRV or influenza infection admitted to the Queen Mary Hospital from 1 March 2014 through 28 February 2015, over a one-year period, were enrolled into a retrospective cohort study, which was approved by the Institutional Review Board of the University of Hong Kong and Hospital Authority Hong Kong West Cluster (IRB reference number: UW 13-265, date of approval 10 April 2013). HRV or influenza infection was confirmed by a positive test by reverse transcription polymerase chain reaction (RT-PCR) of the respective virus on the nasopharyngeal samples (NPS) taken from the patients upon admission [10]. All NPS on admission were assessed by in-house multiplex PCR in the laboratory of the Department of Microbiology, University of Hong Kong, for picornarvirus, EV71, and internal control (bovine viral diarrhea virus). Respiratory specimens were also sent to the Department of Health for detection of influenza A, B, and C viruses, parainfluenza viruses 1, 2, 3, and 4, respiratory syncytial viruses, and adenovirus by three other multiplex PCRs. Bacterial culture in sputum or blood was performed when clinically indicated. Clinical findings including history and physical examination, oximetric measurement, hematological, biochemical, radiological, and microbiological investigation results were retrieved from the clinical management system (CMS) and entered into a predesigned database. Laboratory findings of hemoglobin <13.3 g/dL, neutrophil count >7.42 × 10^9^ /L, lymphocyte count <1.06 × 10^9^ /L, creatinine >109 μmol/L, alaninine transaminase (ALT) >58 U/L, aspartate aminotransferase (AST) >38 U/L, and creatinine kinase >174 IU/L upon admission were defined as abnormal value. Requirement of oxygen therapy, invasive and non-invasive ventilation, intensive care unit (ICU) admission, and hospitalization days and frequency were also assessed.

Comorbidity was categorized into chronic pulmonary diseases, cardiovascular diseases, stroke, malignancy, and diabetes mellitus. Chronic pulmonary diseases include COPD, asthma, and bronchiectasis, whereas cardiovascular diseases include coronary artery disease, valvular heart disease, and congestive heart failure. The chest radiograph findings have been assessed by the clinician in-charge. Patients were followed up from admission for a period of at least 3 months and up to 15 months.

### Statistical Analysis

Clinical and laboratory parameters were compared by γ^2^ for categorical variables and Mann–Whitney *U*-test for continuous variables. Significant risk factors for death were further analyzed by Cox regressions to identify the independent risk factors. A log-rank test was used to evaluate the overall survival over a period of 1-year after admission. IBM SPSS Statistics 10.0 was used for statistical computation. A *p*-value < 0.05 was considered to represent significant difference.

## 5. Conclusions

Limitations of this study include its retrospective nature, and we did not have data on the HRV serotypes in our cohort. In conclusion, HRV infection caused significant mortality and morbidity, especially among those who lived in elderly homes. Infection control measures could reduce HRV infection in these at risk subjects.

## Figures and Tables

**Table 1:** Baseline demographics and laboratory findings of patients hospitalized for rhinovirus infection vs. influenza infection. (*p*-value < 0.05 highlighted in bold).

Baseline Demographics	Rhinovirus (*n* = 728) % (Unless Stated)	Influenza (*n* = 1218) % (Unless Stated)	*p*-Value
Age; Mean (S.D.)	71.6 (20.2)	73.7 (18.6)	0.06
Male sex	44.6	44.7	0.97
Elderly home resident	31.3	23.5	**<0.001**
***Comorbidity***			
Charlson comorbidity index; Mean (S.D.)	0.8 (0.8)	0.8 (0.9)	0.14
Pulmonary diseases	22.9	15.7	**<0.001**
Cardiovascular diseases	21.6	19.9	0.37
Stroke	9.5	13	**0.02**
Malignancy	7.6	6.2	0.22
Diabetes mellitus	26.4	27.7	0.53
Smoker	21.3	19.8	0.35
Influenza vaccination	8.4	9.2	0.54
Pneumococcal vaccination	8.1	12.2	0.06
***Presenting Symptoms***			
Days of symptom onset to admission; Mean (S.D.)	2.8 (4.8)	2.4 (4.2)	0.10
Fever (>37.8)	42.2	65.6	**<0.001**
Cough	57	67.6	**<0.001**
Sputum production	53	49.6	0.15
Sore throat	5.1	14.4	**<0.001**
Chest wheezes	15.4	11.2	**0.007**
Rhinorrhea	6.6	11.2	**0.001**
***Complications***			
Pneumonia	32.6	28	**0.03**
Cardiovascular	3.8	7.6	**0.001**
Sepsis	2.2	4.1	**0.03**
***Treatment***			
Oxygen therapy	33.1	25.4	**<0.001**
Invasive ventilation	0.68	0.74	0.90
Non-invasive ventilation	2.6	3.1	0.52
Hospitalization days; Mean (S.D.)	8.7 (13)	6.8 (12)	**<0.001**
Hospitalization frequency; Mean (S.D.)	2.2 (2.4)	1.9 (1.9)	0.36
ICU admission	1.8	2.4	0.38
***Upon Admission with abnormal value***			
Hemoglobin (<13.3 g/dL)	75.4	69.7	**0.01**
Neutrophil (>7.42 × 10^9^ /L)	42.3	26	**<0.001**
Lymphocyte (<1.06 × 10^9^ /L)	1.8	0.9	0.11
Creatinine (>109 μmol/L)	25.5	27.5	0.37
ALT (>58 U/L)	6	5.7	0.76
AST (>38 U/L)	19.5	28.4	**<0.001**
Creatine kinase (>174 IU/L)	21.8	8.7	**0.01**
***Mortality***			
30-day	9.6	7.1	**0.04**
90-day	14.2	10	**0.006**
1-year	17.2	11.7	**0.004**
***Cause of death***			
Pneumonia	81.6	81.1	0.92
Stroke	3.2	3.5	0.89
Malignancy	10.4	12.7	0.56
Chronic renal failure	2.4	5.6	0.19
Acute myocardial infarction	4.8	2.8	0.66
Congestive heart failure	2.4	5.6	0.19
COPD	20.8	15.4	0.39
Other	1.6	4.2	0.21

**Table 2:** Univariate analysis of factors associated with 1-year mortality in patients hospitalized for rhinovirus infection. (*p*-value < 0.05 highlighted in bold).

Baseline Demographics	Survived (*n* = 603) % (Unless Stated)	Succumbed (*n* = 125) % (Unless Stated)	*p*-Value
Age; Mean (S.D.)	69.4 (20.7)	81.9 (13.6)	**<0.001**
Male sex	45.4	40.8	0.34
Elderly home resident	25.2	60.8	**<0.001**
***Comorbidity***			
Charlson comorbidity index; Mean (S.D.)	0.81 (0.81)	0.95 (0.76)	**0.04**
Pulmonary diseases	21.1	32	**0.008**
Cardiovascular diseases	21.2	23.2	0.63
Stroke	9.1	11.2	0.47
Malignancy	7.1	10.4	0.21
Diabetes mellitus	27	23.2	0.38
Smoker	20.7	24	0.42
Influenza vaccination	8.8	6.4	0.38
Pneumococcal vaccination	11.4	14.4	0.35
***Presenting Symptoms***			
Days of symptom onset to admission; Mean (S.D.)	2.8 (5)	2.7 (4)	0.69
Fever (>37.8)	41.5	45.6	0.39
Cough	55.7	63.2	0.12
Sputum production	48.1	56.8	**0.08**
Sore-throat	7.8	0.8	**0.004**
Chest wheezes	15.1	16.8	0.63
Rhinorrhea	15.6	4	**0.001**
***Complications***			
Pneumonia	27.5	56.8	**<0.001**
Cardiovascular	3	8	**0.008**
Sepsis	1.7	4.8	**0.03**
Bacterial co-infection	1.0	2.4	0.20
***Treatment***			
Oxygen therapy	27	62.4	**<0.001**
Invasive ventilation	0.5	1.6	0.17
Non-invasive ventilation	1.8	6.4	**0.003**
Hospitalization days; Mean (S.D.)	7.6 (11.1)	13.8 (18.6)	**<0.001**
Hospitalization frequency; Mean (S.D.)	2 (2.2)	2.9 (3.4)	**0.02**
ICU admission	1.1	4.8	**0.005**
***Upon Admission with abnormal value***			
Hemoglobin (<13.3 g/dL)	77.6	90.4	**0.001**
Neutrophil (>7.42 × 10^9^ /L)	43	53.6	**0.03**
Lymphocyte (<1.06 × 10^9^ /L)	2.1	0.8	0.37
Creatinine (>109 μmol/L)	25.4	35.2	**0.03**
ALT (>58 U/L)	6.1	8	0.41
AST (>38 U/L)	18.6	30.4	**0.003**
Creatine kinase (>174 IU/L)	9.1	9.6	0.99

**Table 3:** Multivariate analysis of independent risk factors associated with 1-year mortality in patients hospitalized for rhinovirus infection.

Variable	Odds Ratio	95% Confidence Interval	*p*-Value
Rhinorrea	0.31	0.11–0.86	0.024
Elderly home resident	2.60	1.56–4.33	<0.001
ICU admission	9.56	2.17–42.18	0.003
Oxygen therapy	2.62	1.62–4.24	<0.001
Hemoglobin level <13.3 g/dL upon admission	2.43	1.16–5.12	0.019
